# Novel PSMA-Coated On-Off-On Fluorescent Chemosensor Based on Organic Dots with AIEgens for Detection of Copper (II), Iron (III) and Cysteine

**DOI:** 10.3390/polym10070786

**Published:** 2018-07-17

**Authors:** Rui Jiang, Na Liu, Fan Li, Wensheng Fu, Yun Zhou, Yan Zhang

**Affiliations:** Chongqing Key Laboratory of Green Synthesis and Applications, and Chongqing Key Laboratory of Inorganic Functional Materials, College of Chemistry, Chongqing Normal University, Chongqing 401331, China; jiangrui19950213@163.com (R.J.); NaLiunihao@163.com (N.L.); lifan0814@sina.com (F.L.); fuwensheng@cqnu.edu.cn (W.F.)

**Keywords:** fluorescent chemosensor, nanoparticle, aggregation-induced emission, ion detection, cysteine detection

## Abstract

Herein, a novel on-off-on fluorescent chemosensor for copper (II) ion (Cu^2+^), iron (III) ion (Fe^3+^) and cysteine is developed simply by the nano-precipitation method. The prepared organic dots with AIEgens (AIE dots) are advantageous over other metal ions in detecting Cu^2+^, Fe^3+^ with high selectivity and sensitivity by forming agglomerations (on-off). The agglomerations formed by AIE dots and Cu^2+^ redistributed and the fluorescence was obviously recovered in the presence of cysteine (off-on). This sensor has a wide linear range for Cu^2+^, Fe^3+^ and cysteine. The fluorescent detection limits of AIE dots are calculated to be 107 nM for Cu^2+^, 120 nM for Fe^3+^ and 78 nM for cysteine, respectively. These results indicate that the AIE dots can be used as a potential probe for Cu^2+^, Fe^3+^ and cysteine detection.

## 1. Introduction

Different metal ions play very important roles in all organism [1]. Copper (II) iron (Cu^2+^) and iron (III) ion (Fe^3+^) are two of the most significant transition metal ions in physiological and pathological processes, such as redox processes, enzyme-catalyzed reaction, oxygen transport, and electron transport [2,3,4,5]. However, excess Cu^2+^ and Fe^3+^ lead to numerous diseases, such as liver damage, anemia and Alzheimer’s disease [6,7,8]. Moreover, Cu^2+^ and Fe^3+^ cause ecological and environmental problems. Thus, to minimize the harm of excess Cu^2+^ and Fe^3+^, great efforts have been made to develop highly sensitive and selective methods to detect Cu^2+^ and Fe^3+^ [9,10,11,12,13,14].

Cysteine is an essential amino acid, which plays a vital role in organisms in protein synthesis, and metabolism [15,16,17]. However, dysregulation of cysteine in vivo can cause many diseases, such as liver damage, slowed growth, osteoporosis cardiovascular disease [18,19,20]. As a result, quantitative analysis of cysteine is of great importance for the development of biomedicine [21,22].

To date, many sensors with different analysis methods have been reported for chemical analysis [23,24,25,26]. Of these sensors, nanoparticle-based chemical sensors have elicited high interest owing to their simplicity, good sensitivity and selectivity, fast response time, and low cost [27,28]. However, traditional fluorescence materials usually suffer from many disadvantages, which greatly limit their application in chemical detection [29,30]. To avoid the aggregation-caused quenching (ACQ) effect, the concentrations of traditional fluorescence at the nanomolar scale lead to the severe photobleaching effect and low sensitivity [31,32]. In contrast to traditional fluorescence molecules, aggregation-induced emission (AIE) molecules show opposite fluorescence property to ACQ [33], which enables nanoparticles with AIE luminophore to have higher concentrations in order to efficiently improve the photobleaching resistance and detection sensitivity [34,35,36]. These features provide us with an excellent material for chemical sensing. However, very limited efforts have been dedicated to simultaneous detection of Cu^2+^, Fe^3+^ and cysteine by using nanoparticles based on AIE molecules (AIE dots).

In this work, a sensitive “on-off-on” fluorescent sensor for Cu^2+^, Fe^3+^ and cysteine based on AIE dots has been presented. This probe could detect Cu^2+^ and Fe^3+^ by forming agglomerations with obvious fluorescence quenching. By adding cysteine, the agglomerations were redissolved and the fluorescence intensity recovered, due to more stable Cu^2+^–cysteine bond formed. Therefore, this AIE dots could effectively detect Cu^2+^, Fe^3+^ and cysteine.

## 2. Materials and Methods

### 2.1. Materials and Measurements

All the chemicals were purchased from Aladdin (Beijing, China), J&K (Beijing, China) or Sigma-Aldrich (St. Louis, MO, USA), unless specifically stated. All the reagents were commercially available and of analytical reagent grade. The other solvents were purified by fractional distillation. Prior to use, all solvents were purified by fractional distillation. Deionized water with a resistivity of 18.2 MΩ·cm was used from a Milli-Q water purification system (Millipore Corp., Billerica, MA, USA). NMR spectra were recorded by Bruker Avance 300 (Bruker Corp., Rheinstetten, Germany). Mass spectra were recorded on an Agilent 1100 LC-MS system (Agilent Technologies Inc., Foster, CA, USA). The preparation process of AIE dots was completed by a bath sonicator (Bransonic, CPX2800H-C, Emerson Electric Co., Ferguson, MO, USA). UV-Vis absorption spectra were recorded using a UV-2550 UV-Vis spectrophotometer (Shimadzu Corp., Kyoto, Japan). Fluorescence spectra were measured by a Shimadzu RF-5301PC spectrophotometer (Shimadzu Corp., Kyoto, Japan). Transmission electron microscopy (TEM) images of the AIE dots were taken by JEM-2100F at 200 kV (JEOL, Ltd., Kyoto, Japan). DLS measurement was performed using a Malvern ZetasizerNano ZS size analyzer (Malvern Panalytical Ltd., Malvern, UK) at room temperature.

### 2.2. Synthesis of DSA

Synthetic route of DSA was referred to from our previous literature [37,38], and was shown in Scheme S1. Parts of structural characterization data are shown in Supplementary Material.

### 2.3. Preparation of Aggregation-Induced Emission Dots

The AIE dots were prepared for conjugated polymers by using the nano-precipitation method as described previously [39,40]. The tetrahydrofuran stock solutions of PSMA (1 mg·mL^−1^) and DSA (0.5 mg·mL^−1^) were prepared, respectively. Then, a 2-mL DSA solution and different volumes of PSMA solutions were mixed. A one-milliliter mixture was quickly added to 5 mL Milli-Q water and was sonicated in a bath sonicator for one minute. The THF was completely removed by blowing nitrogen. The solution of AIE dots was filtered by a 0.22-micron filter. The maleic anhydride groups of PSMA molecules hydrolyzed into carboxyl during nano-precipitation (as shown in Scheme S2). There was no obvious aggregation of AIE dots at room temperature for months.

### 2.4. Determination of Quantum Yields

By using Coumarine 153 as the reference (*ϕ* = 0.56, an acetone solution with an excitation wavelength of 418 nm), the fluorescence quantum yields of AIE dots with different mass ratios of PSMA to DSA were measured according to the following equation [41]:

$$\varphi_{S}=\varphi_{r}(\frac{A_{r}}{A_{s}})(\frac{I_{s}}{I_{r}})(\frac{\eta_{r}^{2}}{\eta_{s}^{2}})$$

where *ϕ* is the fluorescence quantum yield, *I* is integrated emission intensity, *A* is the absorbance at the excitation wavelength, and *η* is the refractive index of the solvent. The subscripts, *r* and *s*, mean reference and sample, respectively.

### 2.5. Detection of Cu^2+^, Fe^3+^ and Cysteine

For the fluorescence detection, The AIE dots solution was diluted 10 times in a HEPES buffer (2-[4-(2-Hydroxyethyl)-1-piperazinyl]ethane sulfonic acid; pH = 7.4). After ultrasonic washer for 5 min, different metal ions (Ag^+^, Al^3+^, Ba^2+^, Cu^2+^, Ca^2+^, Fe^3+^, Mg^2+^, Mn^2+^, Hg^2+^, K^+^, Na^+^, Pd^2+^, Pb^2+^, Zn^2+^) were added to the solution of AIE dots with the final concentration of 20 μM. The fluorescence spectra were recorded at the excitation wavelength of 406 nm. For comparison, the Cu^2+^ and Fe^3+^ detections were measured in the presence of 100 μM of different metal ions under the same conditions. The effects of different anions on Cu^2+^ detection were examined under the same conditions. The final concentrations of Cu^2+^ and Fe^3+^ ranged from 0 to 30 μM.

For the detection of cysteine, Cu^2+^ with the final concentration of 5 μM was added into a 4-mL buffer solution of AIE dots to get the mixture solution. The fluorescence intensities were measured after different concentrations of cysteine (0 to 4 μM) were added to the mixture solution under ultrasonic irradiation.

## 3. Results and Discussion

### 3.1. Mechanism for the Detection

**Scheme 1:**
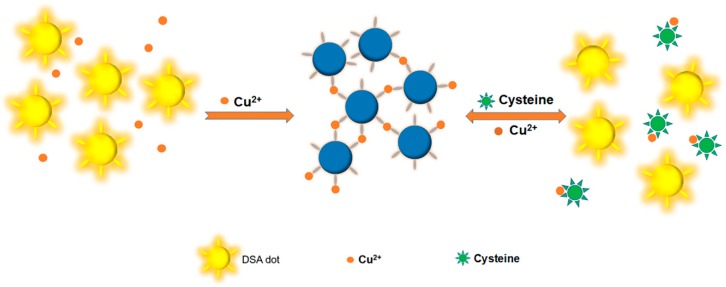
The detection mechanism of AIE dots in the presence of Cu^2+^ and cysteine.

The detection mechanism of AIE dots is shown in Scheme 1. There were a large number of carboxy on the surface of dots, and the AIE dots emitted strong fluorescence under ultraviolet light. The carboxyl served as selective coordination groups for metal ions without further modification. The fluorescence of AIE dots quenched when Cu^2+^ was added into the solution. This quenching was attributed to the agglomeration of AIE dots and the energy-transfer process with the metal ions. Similar agglomeration quenching was also detected in other nano-systems [42]. On the other hand, more stable coordination compound formed and the agglomerations of AIE dots could be redissolved, when cysteine was added into the suspension. With the agglomerations disassociated, the “turn-on” fluorescence was observed.

### 3.2. Preparation and Characterization of Aggregation-Induced Emission Dots Based Probe

Due to the high fluorescence quantum yields (*ϕ* = 0.40) and AIE property [43], DSA is a suitable material for preparing organic dots. In this work, PSMA was used as a packed material and DSA was employed as a luminescent material. After AIE dots were prepared in aqueous environment, the hydrophobic groups (such as benzene and DSA) were encapsulated inside the dots, and carboxy groups, which hydrolyzed from maleic anhydride groups, and were distributed on the surface of AIE dots, as shown in Scheme S2. By regulating the mass ratio of DSA to PSMA in the precursor solution (from 1:4 to 2:1), four types of AIE dots were obtained. The DLS data showed that all the AIE dots had a narrow particle size of around 23 nm, as shown in Figure S1.

**Figure 1:**
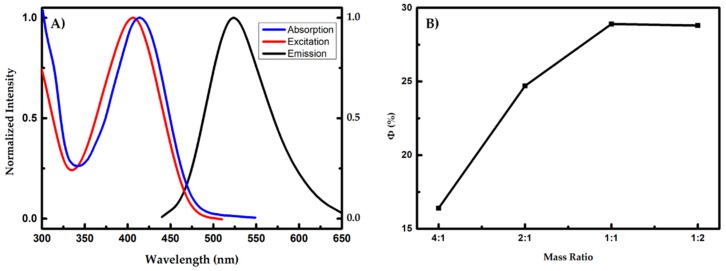
(**A**) Absorption, excitation, and emission spectra of AIE dots; (**B**) the fluorescence quantum yield of AIE dots as a function of the mass ratio between PSMA and DSA (PSMA:DSA = 4:1, 2:1, 1:1, 1:2).

The aqueous solutions of AIE dots were light green colloidal solutions and the solutions emitted strong green-yellow fluorescence under an ultraviolet lamp with the excitation wavelength of 365 nm. The absorption spectra, excitation and fluorescence spectra of AIE dots are shown in Figure 1A. From the absorption spectra, the maximum absorption wavelength of AIE dots with different mass ratios of PSMA to DSA was approximately 414 nm. This absorption was similar to the absorption of the DSA molecule, which could be originated from the π–π* transition [43,44]. The maximum emission peak of all AIE dots was approximately 524 nm and the maximum excitation wavelength of all AIE dots were approximately 406 nm. After overall consideration, we chose 406 nm as the excitation wavelength in the following detection.

The fluorescence quantum yields of AIE dots with different mass ratios of PSMA to DSA were measured. The fluorescence quantum yields was increased as the concentration of dye increased, as shown in Figure 1B. After the mass ratio of PSAM to DSA reached 1:1, the fluorescence quantum yields of AIE dots was no longer rising. The fluorescence quantum yield of PSMA dots was 0.29, when the mass ratio of PSMA to DSA was 1:1. Therefore, the following detection was experimented for the mass ratio between PSMA and DSA of 1:1.

### 3.3. Aggregation-Induced Emission Dots for Recognition of Cu^2+^ and Fe^3+^

**Figure 2:**
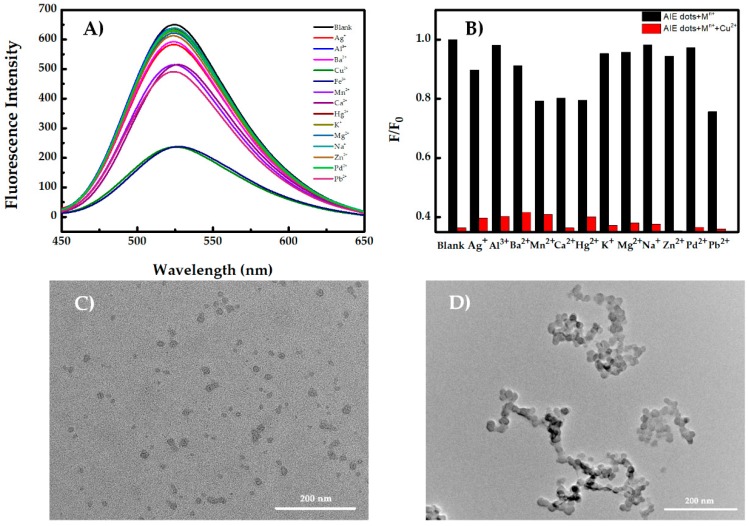
(**A**) Fluorescence spectra of AIE dots in the presence of different metal ions (20 μM). (**B**) Selective fluorescence responses of the Cu^2+^ system in the presence of other metal ions. The black bars represent the emission in the presence of different ions with a concentration of 0.1 mM. The red bars represent the emission in the presence of 20 μM Cu^2+^ and another metal ion with a concentration of 0.1 mM. *F*_0_ and *F* correspond to the fluorescence intensity at 524 nm in the absence and the presence of Cu^2+^, respectively. (**C**) TEM image of AIE dots. (**D**) TEM image of AIE dots with Cu^2+^ (20 μM).

Figure 2A shows that most of the tested metal ions only induced minor fluorescence quenching, while Cu^2+^ and Fe^3+^ could significantly quench the fluorescence intensity. Cu^2+^ and Fe^3+^ with a concentration of 20 μM caused 63% fluorescence quenching, which was much higher than other metal ions. This result indicated that AIE dots had excellent selectivity for Cu^2+^ and Fe^3+^. In addition, the interfering effects from other metal ions (0.1 mM) were measured (Figure 2B for Cu^2+^ and Figure S2 for Fe^3+^). No obvious interference with Cu^2+^ and Fe^3+^ was found when they coexisted with other metal ions. In addition, Cu^+^ and Fe^2+^ with a concentration of 20 μM had only small interference (Figure S3). Meanwhile, different anions did not have any interference for the detection of Cu^2+^, as shown in Figure S4. The interfering effects from high concentrations of Mg^2+^, K^+^ and Na^+^ were measured as well (Figure S5), and there was not any significant effect on detection of Cu^2+^ and Fe^3+^. These results further confirmed that AIE dots could act as a sensitive Cu^2+^/Fe^3+^ fluorescence sensor and were expected to detect Cu^2+^/Fe^3+^ selectively in complicated biological environment.

The agglomeration and self-quenching behavior of AIE dots was attributed to the chelating interactions between the carboxyl with Cu^2+^ or Fe^3+^ in the solution. Take Cu^2+^ as an example. The AIE dots and agglomeration were measured by DLS (Figure S6A,B) and TEM (Figure 2C,D). The DLS measurements showed that the diameter of the AIE dots was 23 nm with a narrow particle size distribution, but the particle size increased to about 400 nm after Cu^2+^ was added. This phenomenon was clearly presented in the TEM images taken before and after the addition of Cu^2+^. The similar results could be found after Fe^3+^ was added, as shown in Figures S6C and S7A.

**Figure 3:**
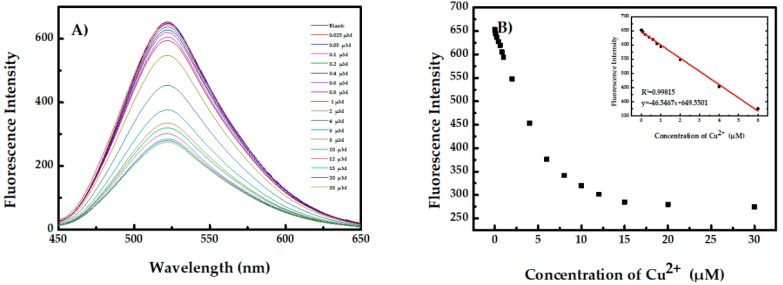
(**A**) Fluorescence spectra of AIE dots in the presence of different concentrations of Cu^2+^ excited at 406 nm. (**B**) The plot of the fluorescence intensity ratio of AIE dots at 524 nm versus different concentrations of Cu^2+^; the inset shows the signal change in the Cu^2+^ concentration range of 0–6 μM.

The fluorescence quenching response of AIE dots with different concentrations of Cu^2+^ in the titration process was shown in Figure 3A. As the concentration of Cu^2+^ increased, the fluorescence intensity of AIE dots around 524 nm decreased continuously. The linear range of Cu^2+^ was from 0.025 to 6 μM (*R*^2^ = 0.99815), which was a wide concentration range (Figure 3B). The fluorescence detection limit of AIE dots for Cu^2+^ was estimated to be 107 nM, which was much lower than the standard of drinking water and food (20 and 5.4 μM, respectively) set by Environmental Protection Agency (EPA) of the USA government [45]. Similarly, the detection limit of Fe^3+^ was 120 nM (Figure S8). These results demonstrated that AIE dots could be employed as Cu^2+^/Fe^3+^ sensors with excellent selectively and sensitivity.

### 3.4. Detection of Cysteine

**Figure 4:**
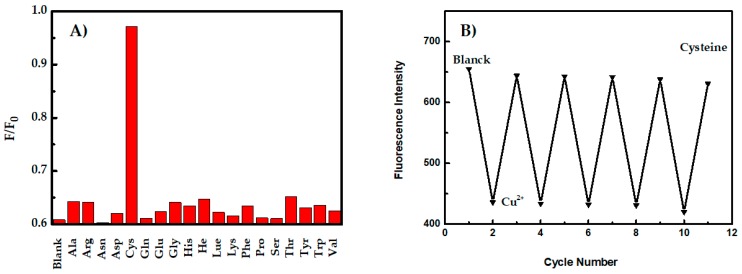
(**A**) Fluorescence changes of AIE dots/Cu^2+^ in the presence of different amino acids with a concentration of 5 μM. (**B**) Fluorescence intensity as a function of the cycle number. The fluorescence intensity of AIE dots after quenching by Cu^2+^ (5 μM) can be recovered by the addition of 2.5 μM cysteine.

Thiols have a strong interaction with Cu^2+^ and form the Cu^2+^–S bond. Therefore, biothiols could eliminate the coupling effect between Cu^2+^ and AIE dots and recover the fluorescence of AIE dots in this system. For detecting of biothiols, cysteine was added to the AIE dots/Cu^2+^ aggregation suspension, and the effect of other amino acids was evaluated. As shown in Figure 4A, cysteine led to nearly 97% fluorescence recovery while only a little recovery for the other amino acids was observed. In order to determine whether Fe^3+^ could block the fluorescence recovery, spike recovery of Cu^2+^, Fe^3+^ and cysteine was carried out (Figure S10A). The fluorescence could not restore when Cu^2+^ and Fe^3+^ coexisted. To eliminate the effect of Fe^3+^, F^−^ was used to coordinate Fe^3+^. As shown in Figure S10B, the interference of Fe^3+^ was blocked when F^−^ was added, and the fluorescence recovered after cysteine was added. These results indicated that AIE dots/Cu^2+^ suspension could serve as a good turn-on sensor for detecting cysteine.

The fluorescence enhancement effect was measured in the presence of different concentrations of cysteine (Figure S9A). The fluorescence intensity of AIE dots gradually recovered as the concentration of cysteine increased. The linear range of cysteine was from 0.025 to 2.5 μM (Figure S9B inset). The fluorescence detection limit for cysteine was estimated to be 78 nM based on three times the standard deviation rule. Cu^2+^ (5 μM) or cysteine (2.5 μM) was added into the solution of AIE dots 5 times, as shown in Figure 4B. The fluorescence quenched when Cu^2+^ was added, and fluorescence recovered after cysteine was added, as a result of the more stable Cu^2+^–S bond formed and the AIE dots released (Figures S6D and S7B). This repeatability of the emission recovery demonstrated the excellent reliability of this probe.

## 4. Conclusions

In summary, a novel fluorescence dots based sensor (AIE dots) with a surfactant (PSMA) was developed for “on-off-on” detection of Cu^2+^, Fe^3+^ and cysteine. Based on the agglomeration of AIE dots, a turn-off detection for Cu^2+^ and Fe^3+^ could be realized. Meanwhile, fluorescence recovered when Cu^2+^–cysteine chelates was formed. The linear detection range for Cu^2+^, Fe^3+^ and cysteine was within the physiologically relevant concentration range. However, this linear range could be further extended, if needed. The fluorescent detection limits for Cu^2+^, Fe^3+^ and cysteine were measured to be 107, 120 and 78 nM, respectively. The excellent performance of this fluorescence probe affords a means of rapid determination of Cu^2+^, Fe^3+^ and cysteine in environmental and biological applications.

## Supplementary Materials

Supplementary Materials are available online at http://www.mdpi.com/2073-4360/10/7/786/s1.
