# Supplementary material for: Novel PSMA-Coated On-Off-On Fluorescent Chemosensor Based on Organic Dots with AIEgens for Detection of Copper (II), Iron (III) and Cysteine
Source: Polymers (Basel). 2018 Jul 17;10(7):786. doi: 10.3390/polym10070786 (PMC6403782; doi:10.3390/polym10070786)
Supplement: Supplementary file 1 [file polymers-10-00786-s001.pdf]

Novel PSMA-Coated on-off-on Fluorescent Chemosensor based on AIE Dots for Detection of Copper (II) 、Iron (III) and Cysteine.

Rui Jiang, Na Liu, Fan Li, Wensheng Fu, Yun Zhou* and Yan Zhang*

Chongqing Key Laboratory of Green Synthesis and Applications, and Chongqing Key Laboratory of Inorganic Functional Materials, College of Chemistry, Chongqing Normal University, Chongqing 401331, China; jiangrui19950213@163.com (R.J); NaLiunihao@163.com (N.L); lifan0814@sina.com (F.L); fuwensheng@cqnu.edu.cn (W.F.)

* Correspondence: yanzhang@cqnu.edu.cn; yunzhou@cqnu.edu.cn; Tel.: +86-23- 6536-2777

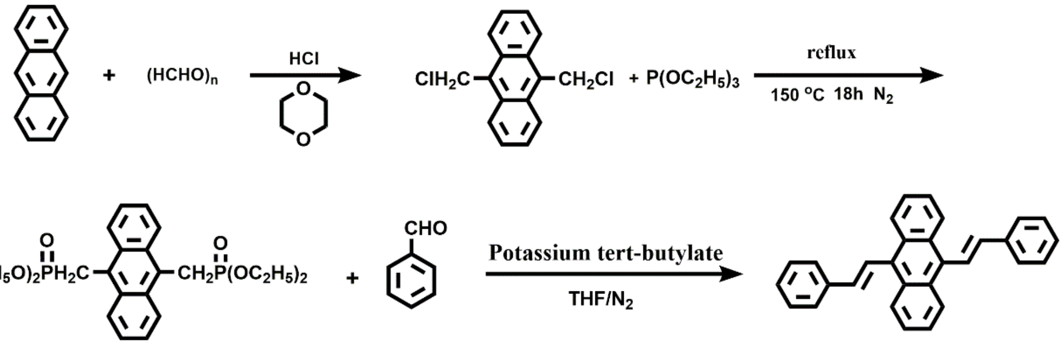

Scheme S1. Synthetic route of DSA

The characterization of DSA:^{1}H NMR (100 MHz, $CDCl_3$, TMS, ppm): 6.95 (d, 2H), 7.37 (t, 2H), 7.46-7.49 (m, 8H), 7.70 (d, 4H), 7.94 (d, 2H), 8.39-8.41 (m, 4H). ^{13}C NMR (25 MHz, $CDCl_3$, TMS, ppm): 137.46, 137.28, 132.68, 129.56, 128.84, 128.03, 126.60, 126.46, 125.24, 125.16. LC-MS calcd for $C_{30}H_{22}$ 382.2, found 382.6.

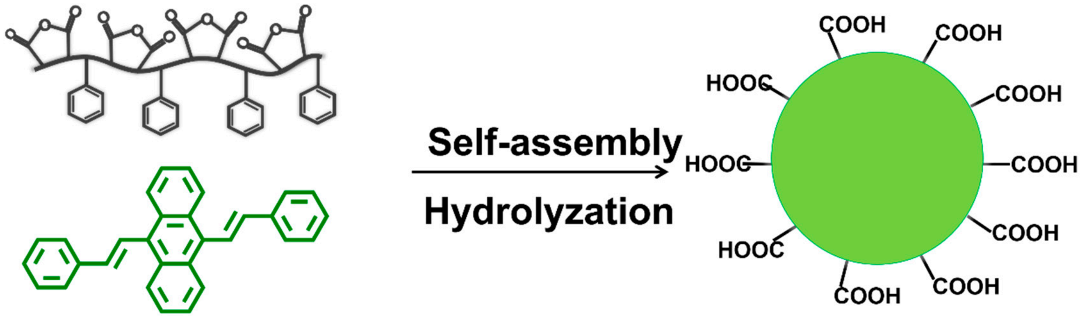

Scheme S2. The preparation route of AIE dots.

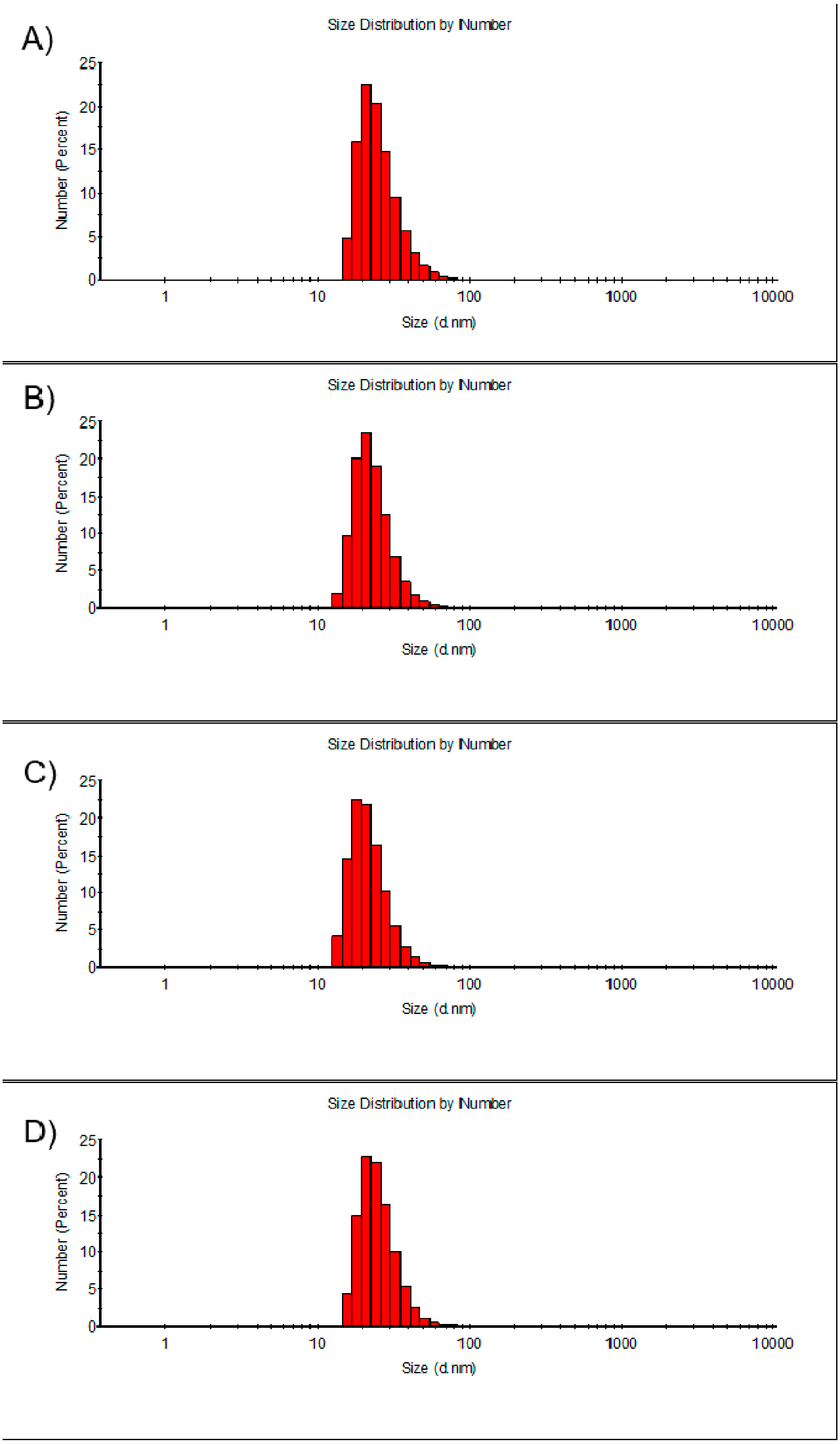

Figure S1. DLS data of AIE dots prepared with different mass ratio (from A to B, PSMA: DSA = 4:1, 2:1, 1:1, 1:2).

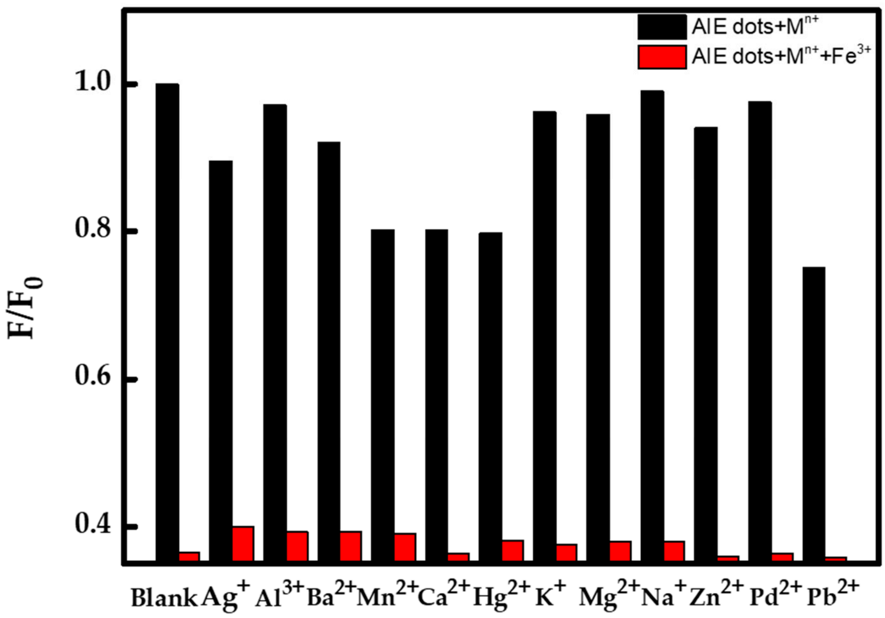

Figure S2. Selective fluorescence responses of Fe^{3+} system in the presence of other metal ions. The black bars represent the emission in presence of 0.1 mM different ions. The red bars represent the emission in the presence of 20 μM Fe^{3+} and 0.1 mM another metal ion.

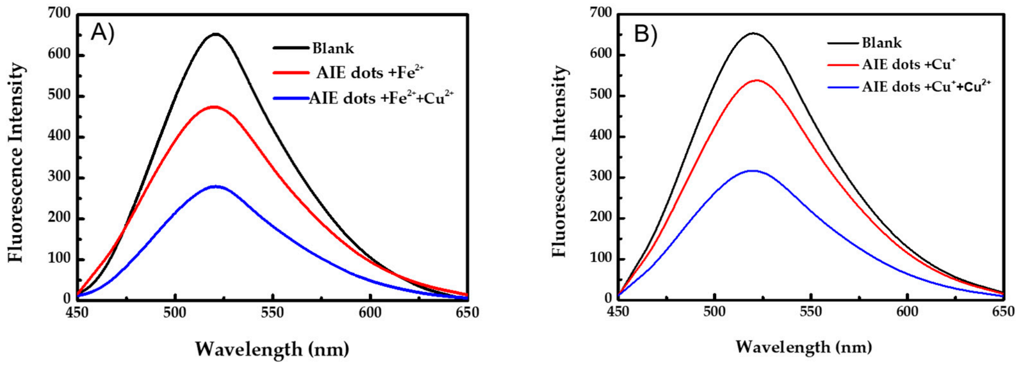

Figure S3. Selective fluorescence responses of Cu^{2+} system in the presence of A) Fe^{2+} and B) Cu^{+} (20 μM).

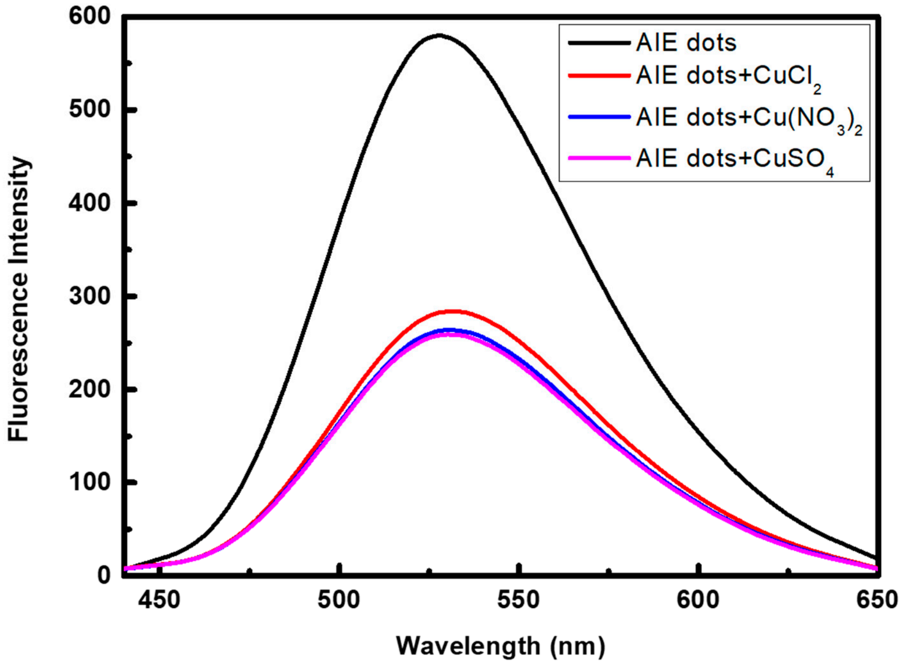

Figure S4. Effects of different anions (Cl^{-}, NO_3^{-}, SO_4^{2-}). The concentration of Cu^{2+} was 20 μM.

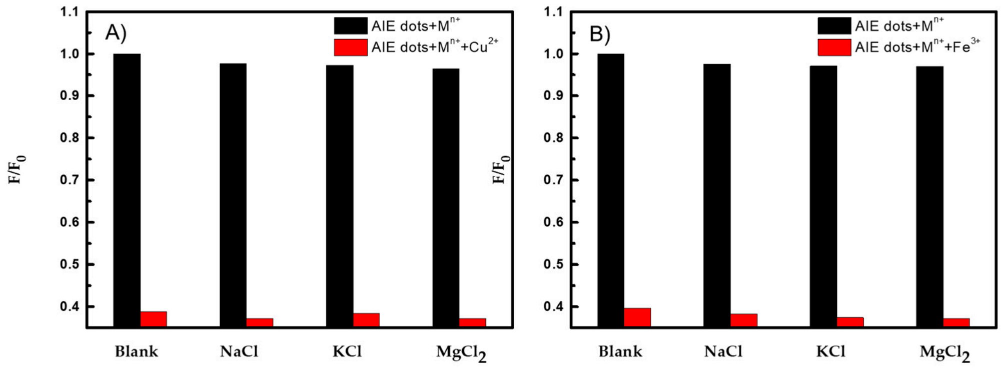

Figure S5. Selective fluorescence responses of A) Cu^{2+} and B) Fe^{3+} system (20 μM) in the presence of high concentration of Mg^{2+}, K^{+}, and Na^{+} (2nM, 6mM, 150 mM, respectively).

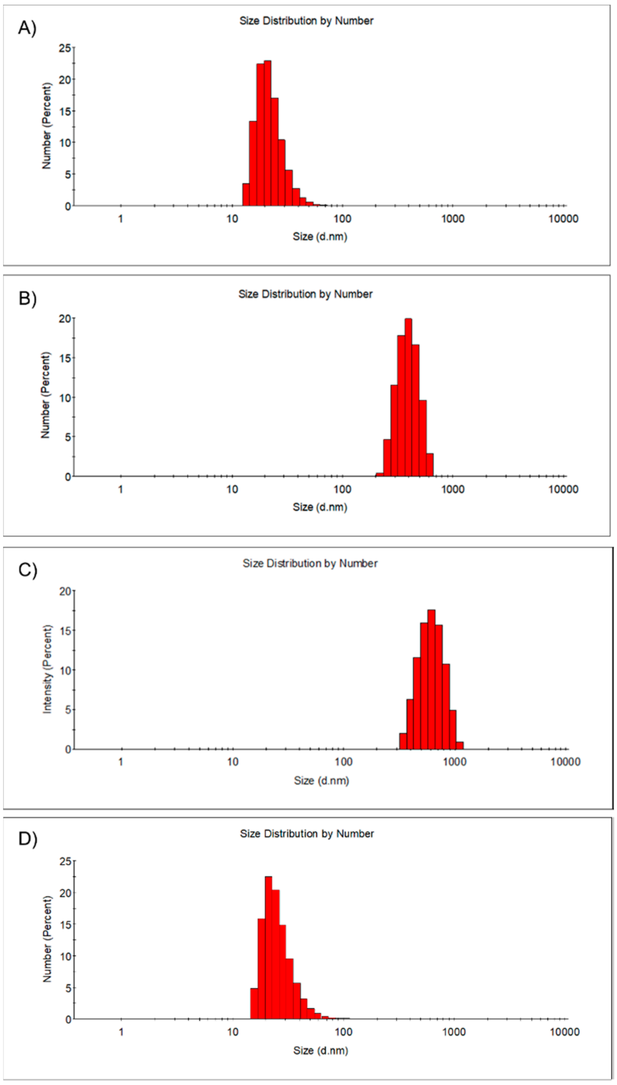

Figure S6. A) The DLS of AIE dots. B) The DLS of AIE dots with Cu^{2+} (20 μM). C) The DLS of AIE dots with Fe^{3+} (20 μM). D) The DLS of AIE dots with Cu^{2+} (5 μM) and cysteine (2.5 μM).

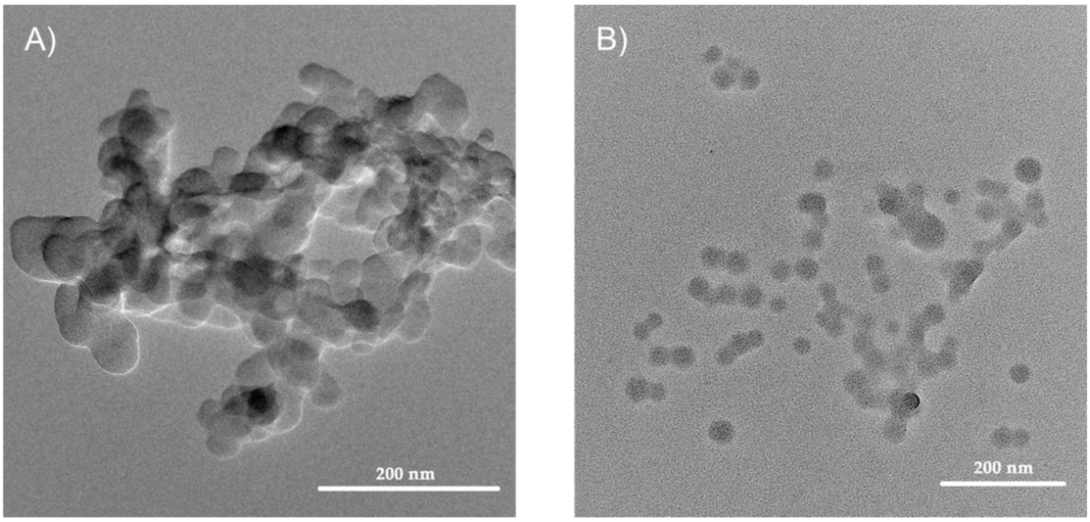

Figure S7 A) The TEM images of AIE dots with Fe^{3+}. B) The TEM images of AIE dots/Cu^{2+} in the presence of cysteine.

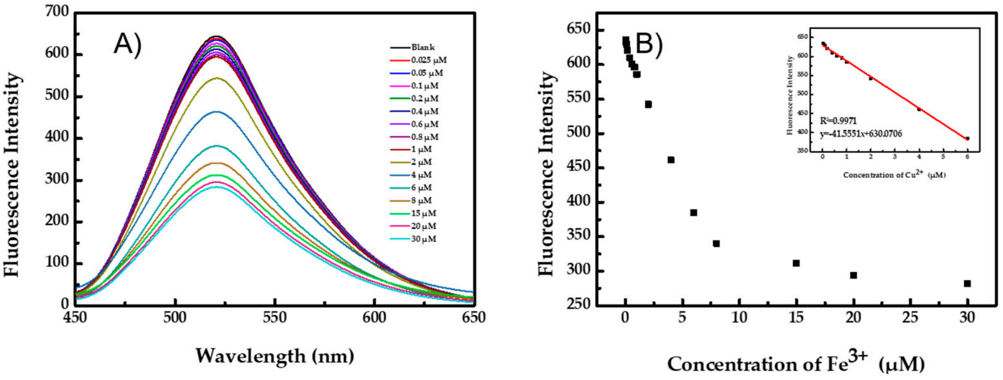

Figure S8. A) Fluorescence changes of AIE dots in the presence of different concentration of Fe^{3+} excited by 406 nm. B) The plot of the fluorescence intensity ratio of AIE dots at 524 nm versus different concentration of Fe^{3+}. The inset figure shows the signal change in the Fe^{3+} concentration range of 0-6 μM.

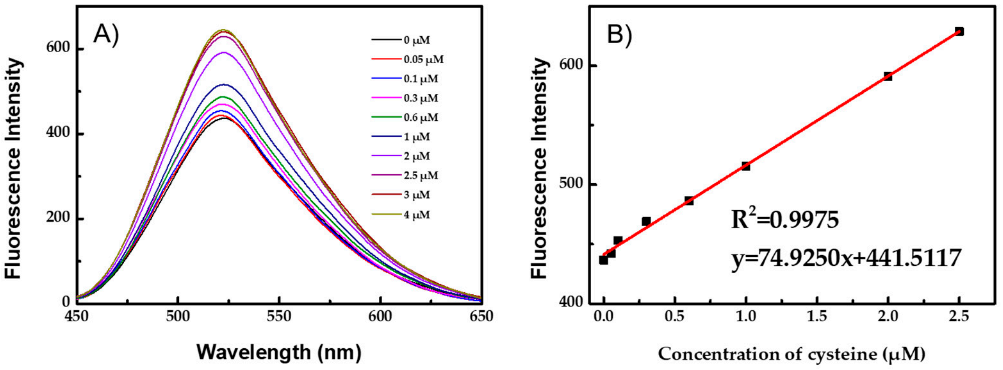

Figure S9. A) Fluorescence intensity changes of AIE dots containing Cu^{2+} (5 μM) in the presence of different concentrations of cysteine (0-4 μM). B) The plot of the fluorescence intensity ratio of AIE dots/Cu^{2+} at 524 nm versus different concentration of cysteine (0-2.5 μM).

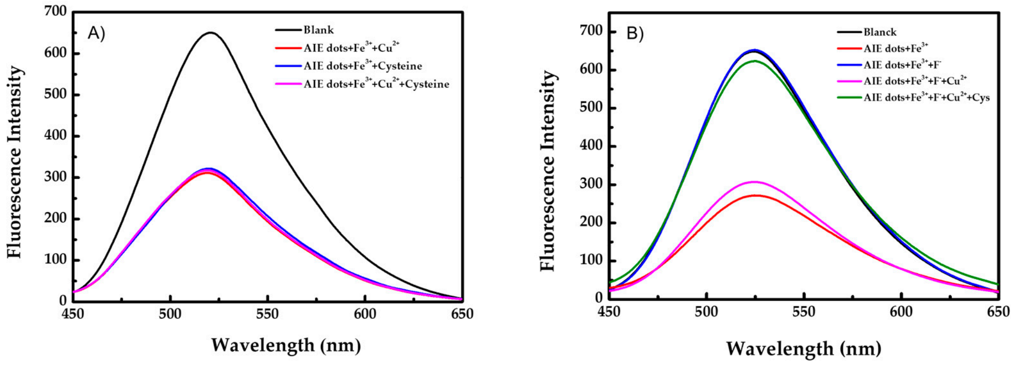

Figure S10. A) Spike recovery of Cu^{2+}, Fe^{3+} and cysteine. B) Fluorescence intensity changes of AIE dots in the presence of Fe^{3+}, Fe^{3+} & F^-, Fe^{3+} & F^- & Cu^{2+}, Fe^{3+} & F^- & Cu^{2+} & cysteine.
